# FDG-PET/CT Demonstrates Superior Detection of Multiorgan Involvement in Paracoccidioidomycosis Compared With Conventional Staging

**DOI:** 10.1093/cid/ciag372

**Published:** 2026-06-19

**Authors:** Pricila G Cunha, Maria Emilia Seren Takahashi, Raquel S B Stucchi, Mariana Paixão Ribeiro, Thiago F Souza, Carmino Antonio de Souza, Barbara Juarez Amorim, Maria Luiza Moretti, Plinio Trabasso, Celso Dario Ramos

**Affiliations:** Division of Nuclear Medicine, Department of Radiology and Oncology, School of Medical Sciences, University of Campinas (UNICAMP), Campinas, Brazil; Division of Nuclear Medicine, Department of Radiology and Oncology, School of Medical Sciences, University of Campinas (UNICAMP), Campinas, Brazil; Gleb Wataghin Institute of Physics, University of Campinas (UNICAMP), Campinas, Brazil; Division of Infectious Diseases, Department of Internal Medicine, School of Medical Sciences, University of Campinas (UNICAMP), Campinas, Brazil; Division of Nuclear Medicine, Department of Radiology and Oncology, School of Medical Sciences, University of Campinas (UNICAMP), Campinas, Brazil; Division of Nuclear Medicine, Department of Radiology and Oncology, School of Medical Sciences, University of Campinas (UNICAMP), Campinas, Brazil; Center of Hematology and Hemotherapy, University of Campinas (UNICAMP), Campinas, Brazil; Division of Nuclear Medicine, Department of Radiology and Oncology, School of Medical Sciences, University of Campinas (UNICAMP), Campinas, Brazil; Division of Infectious Diseases, Department of Internal Medicine, School of Medical Sciences, University of Campinas (UNICAMP), Campinas, Brazil; Division of Infectious Diseases, Department of Internal Medicine, School of Medical Sciences, University of Campinas (UNICAMP), Campinas, Brazil; Division of Nuclear Medicine, Department of Radiology and Oncology, School of Medical Sciences, University of Campinas (UNICAMP), Campinas, Brazil

**Keywords:** Paracoccidioidomycosis, FDG-PET/CT, Fungal diseases, Neglected tropical diseases, Adrenal involvement

## Abstract

**Background:**

Paracoccidioidomycosis (PCM) is a neglected systemic mycosis in which accurate staging is critical but challenging with conventional methods. This study evaluated ^18^F-FDG PET/CT compared with standard evaluation for PCM staging and assessed its impact on disease severity classification.

**Methods:**

Thirty-four patients with confirmed PCM (treatment-naïve or with suspected treatment failure) underwent conventional staging and whole-body ^18^F-FDG PET/CT. Disease severity was classified as mild, moderate, or severe. PET/CT images were analyzed qualitatively and quantitatively (SUVmax, TMLV, TLG).

**Results:**

PET/CT detected more than twice as many lesions as conventional evaluation (150 vs 62; mean 4.4 ± 1.6 vs 1.8 ± 0.9 affected organ systems per patient), with statistically significant superiority for lymph node, pulmonary, and adrenal involvement (McNemar's test, *P* < .05). PET/CT reclassified 97% of patients as multifocal versus 59% by conventional staging and produced a significant unidirectional shift toward higher severity—the proportion classified as severe increased from 70.6% to 91.2% (*P* = .016). Patients with suspected treatment failure showed significantly lower TMLV and TLG than treatment-naïve patients (*P* < .05). Quantitative PET parameters correlated with serological titers and inflammatory markers (*P* < .05), and PET/CT detected four times as many sites as gallium-67 scintigraphy.

**Conclusions:**

^18^F-FDG PET/CT systematically reveals greater disease burden than conventional evaluation in PCM, reclassifying most patients toward higher severity with direct implications for treatment duration and follow-up. Where available, it should be considered an important complementary modality for initial disease assessment.

## Teaser

FDG-PET/CT detects far more lesions than conventional methods in PCM, reclassifying most patients as multifocal and revealing extensive occult involvement. Quantitative PET metrics correlate with disease activity, supporting its value for accurate staging and potential use in treatment monitoring.

Paracoccidioidomycosis (PCM) is a systemic fungal infection caused by species of the *Paracoccidioides* genus, recently reclassified into five phylogenetic species [1,2]. The disease can involve virtually any organ, spreading initially via the lymphatic system and later hematogenously [3,4]. Diagnosis relies on the direct identification of *Paracoccidioides* spp. in clinical or histopathological samples, with serological assays providing complementary information for diagnosis and treatment monitoring [5]. It is a neglected tropical disease with significant morbidity and mortality, ranking eighth among chronic infectious diseases in terms of deaths and presenting the highest lethality among systemic mycoses [5–8]. Disease staging is essential for determining treatment duration, prognosis, and prevention of sequelae [9].

Paracoccidioidomycosis is classified into three main categories: infection, active disease, and residual forms (sequelae). Active disease encompasses the acute/subacute (juvenile) form—graded as moderate or severe—and the chronic (adult) form, which is further stratified by severity (mild, moderate, or severe) and anatomical distribution (unifocal or multifocal). This classification, originally proposed by Franco et al [10] and refined by subsequent Brazilian and international guidelines [5], is based on clinical and conventional imaging evaluation, which may substantially underestimate the true extent of systemic involvement. Notably, the recently introduced concept of an isolated organic form differs from a simplistic unifocal designation, as it requires systematic exclusion of classically affected organs [4, 11, 12]. The accuracy of any unifocal or multifocal classification, therefore, is inherently dependent on the sensitivity of the staging modality employed—a limitation that whole-body functional imaging may help overcome.

Currently, staging relies on clinical examination, serology, and chest radiography, which often identifies pulmonary disease as the sole manifestation (reported in ∼25% of patients) [9, 13]. Additional imaging—such as ultrasound, computed tomography (CT), magnetic resonance imaging (MRI), or gallium-67 scintigraphy—is usually performed only in selected cases with suspected multifocal involvement [5, 9, 14, 15]. However, a sensitive whole-body imaging modality could improve staging, guide therapy, and support earlier detection of systemic disease.

Over recent decades, ^18^F-FDG PET/CT has emerged as a cornerstone in oncology [16, 17] and as a valuable tool in infectious and inflammatory diseases [18, 19]. Compared with gallium-67 scintigraphy, PET/CT provides superior anatomical resolution, faster acquisition, and the ability to quantify metabolic activity using standardized uptake value (SUV), metabolic lesion volume (MLV), and total lesion glycolysis (TLG) [20]. PET/CT has been applied to fungal infections [21, 22], but its role in PCM remains unexplored.

This study aimed to evaluate the diagnostic performance and potential clinical utility of FDG-PET/CT in PCM, compared with conventional evaluation.

## METHODS

### Study Design and Population

This single-center, prospective, observational study was approved by the local Research Ethics Committee (CAE 19682213.0.000005404). All participants provided written informed consent. We enrolled 34 patients with a microbiological or histopathological diagnosis of PCM who were either treatment-naïve (less than 2 months of therapy) or on prolonged treatment with suspected clinical or serological failure. Pregnant or lactating women were excluded.

### Conventional Staging Evaluation

All patients underwent a standardized assessment per Brazilian PCM management guidelines [5]. This included a detailed clinical examination, serological testing via double immunodiffusion, and measurement of inflammatory markers (C-reactive protein [CRP] and erythrocyte sedimentation rate [ESR]). Conventional imaging (chest X-ray, CT, MRI, abdominal ultrasound, gallium-67 scintigraphy, bone scintigraphy) was performed based on clinical suspicion of multifocal disease. All sites of involvement identified by these methods were recorded.

Three patients presented with the acute/subacute (juvenile) form of PCM—all classified as severe—while the remaining 31 had the chronic (adult) form. None of the patients met the criteria for the isolated organic form. Overall, 5 patients (14.7%) were classified as mild, 5 (14.7%) as moderate, and 24 (70.6%) as severe, including the 3 patients with the juvenile form.

### FDG-PET/CT Acquisition Protocol

Patients fasted for at least 6 h prior to intravenous administration of 4.4 MBq/kg of FDG, provided blood glucose was <180 mg/dL. Whole-body PET/CT images were acquired 60 min postinjection using a Siemens Biograph True-Point mCT 40 scanner (Siemens Medical Solutions, Inc., Knoxville, Tennessee, USA). Low-dose CT for attenuation correction and anatomical localization was performed without intravenous contrast (120–140 kV, 120 mA, rotation time 0.8 s, and slice thickness 2.1 mm). PET images were reconstructed using an iterative algorithm (3D-OSEM + PSF + TOF with 2 iterations and 21 subsets).

### Qualitative Image Analysis

PET/CT images were independently analyzed by two experienced nuclear medicine physicians and one radiologist, all blinded to the results of other imaging studies as well as to clinical and laboratory data, including serological titers and inflammatory markers. Readings were performed by consensus. The number and location of regions with abnormal tracer uptake were recorded. Lymph node chains were categorized into the following groups: cervical, axillary, thoracic, abdominal, and pelvic/inguinal. Gastrointestinal tract lesions were considered positive only when uptake was focal and clearly exceeding physiological activity.

### FDG-PET/CT Image Quantification

Maximum SUV (SUVmax), defined as the highest SUV found in a volume of interest, was calculated for each lesion. Total MLV (TMLV), defined as the sum of the volumes of all active lesions, was calculated for all patients using the Beth Israel Plugin for Fiji [23], with a threshold SUV of 2.5. Total lesion glycolysis (TLG), which takes into account both the volumes and uptake intensities of the lesions, was obtained by multiplying the MLV of each lesion by its mean SUV.

### FDG-PET/CT Dual-Time-Point Imaging

To assess the potential utility of dual-time-point imaging (DTPI) [24] for characterizing PCM lesions, additional delayed acquisitions were performed 2 h after radiopharmaceutical injection. The SUVmax of the most active lesions on the early PET/CT scan (SUVmaxE) was also measured on the delayed scan (SUVmaxD). The percentage variation in SUV (SUVvar) was calculated as follows: SUVvar (%) = [(SUVmaxD − SUVmaxE)/SUVmaxE] × 100.

### Comparison With Gallium-67 Scintigraphy

In cases where gallium-67 scintigraphy was clinically indicated for suspected multiorgan involvement, images were acquired following state-of-the-art protocols to allow direct comparison with FDG-PET/CT. Gallium-67 scintigraphy was performed on a dual-head SPECT/CT gamma camera (Symbia T2, Siemens Medical Solutions, Inc., Knoxville, TN, USA). Images were obtained 48 h after intravenous administration of 111–185 MBq of ^67^Ga-citrate. Acquisition was performed using a large field-of-view camera with a medium-energy collimator and triple-energy window settings (93, 184, and 296 keV). Additional SPECT/CT images of the thorax and abdomen were acquired (128 × 128 matrix, 60 projections over 360°, 20 s/projection). Computed tomography parameters included 130 kV, 60 mAs, 0.8 s rotation time, and 3 mm slice thickness. All scans were reviewed by consensus between two nuclear medicine physicians and one radiologist.

### Statistical Analysis

Categorical variables were summarized as absolute counts (n) and percentages (%). Numerical variables were expressed as mean, standard deviation, and minimum and maximum values. Spearman's correlation coefficient was used to assess the association between numerical variables. The Mann–Whitney U test and Kruskal–Wallis test were applied to evaluate the relationship between serological test results (categorical) and SUV, TMLV, and TLG. Categorical variables presented as proportions were analyzed using the chi-squared test or Fisher's exact test, as appropriate. McNemar's test was used to compare paired categorical results between PET and the conventional examination for each anatomical site. The quadratic weighted kappa statistic was used to assess agreement between disease severity classifications based on clinical criteria alone and those incorporating both clinical criteria and PET/CT imaging findings. A significance level of 5% (*P* < .05) was adopted for all analyses. Statistical analyses were performed using SAS software for Windows, version 9.4 (SAS Institute, Inc., Cary, NC, USA; 2002–2008).

## RESULTS

### Patients

Thirty-four patients (23 males, mean age 49.2 ± 13.6 years, range 23–73) with confirmed PCM were included. Twenty-two had received <2 months of treatment (median 15.5 days), and 12 had received >2 months (median 124 days) but were suspected of treatment failure. Three patients had the juvenile form. Serological testing for PCM could not be performed in one patient. Demographic data are summarized in Table 1.

**Table 1.** Patient Characteristics

Number of patients [n]	34
**Sex [n (%)]**	
Male	23 (67.6%)
Female	11 (32.4%)
**Age [y]**	
Mean ± SD	49.2 ± 13.6
Median (max–min)	51.5 (23.0–73.0)
**Patient presentation [n (%)]**	
Initial evaluation^a^	22 (64.7%)
Suspected treatment failure	12 (35.3%)
**Serology**	
N	33
Negative, [n (%)]	8 (24.2%)
Mildly abnormal^b^, [n (%)]	20 (60.6%)
Moderate/severely abnormal^c^, [n (%)]	5 (15.2%)
**CRP**	
Mean ± SD	13.5 ± 19.9
Median (max–min)	5.31 (0.10–89.70)
**ESR**	
Mean ± SD	49.9 ± 39.1
Median (max–min)	33.0 (2.0–120.0)
**Drug treatment** n (%)	
Sulfamethoxazole	10 (29.4%)
Itraconazole	13 (38.2%)
Drug associations	11 (32.4%)
**Comorbidities [n=15]** n (%)**^d^**	
COPD	8 (53.3%)
Diabetes	6 (40.0%)
Renal failure	3 (20.0%)
Other immunodepressions	3 (20.0%)

COPD, chronic obstructive pulmonary disease; CRP, C-reactive protein; ESR, erythrocyte sedimentation rate; SD, standard deviation.

^a^ <2 m after initial presentation.

^b^Antibody titers from 1:2 to 1:8.

^c^Antibody titers from 1:16 to 1:256.

^d^Some had more than one comorbidity, so totals exceed 100%.

### Conventional Disease Staging vs PET/CT

Chest radiography was the most commonly used conventional image (used in 25 patients), followed by CT (12 patients), gallium-67 scintigraphy (8 patients), MRI (3 patients), abdominal US (1 patient), and bone scintigraphy (1 patient).

Conventional evaluation classified 20 of 34 patients (59%) as multifocal—15 with only two foci—and 14 of 34 (41%) as unifocal. FDG-PET/CT reclassified 33 of 34 (97%) as multifocal—2 with two foci—with just 1 case (3%) unifocal (Figure 1). Overall, PET/CT detected 150 lesions compared with 62 by conventional methods (mean 4.4 ± 1.6 vs 1.8 ± 0.9 affected areas per patient). PET/CT identified additional lesions in lymph nodes, lungs, adrenal glands, bone, subcutaneous tissue, gastrointestinal tract, thyroid, and testes (Table 2, Figures 2 and 3).

**Figure 1.**
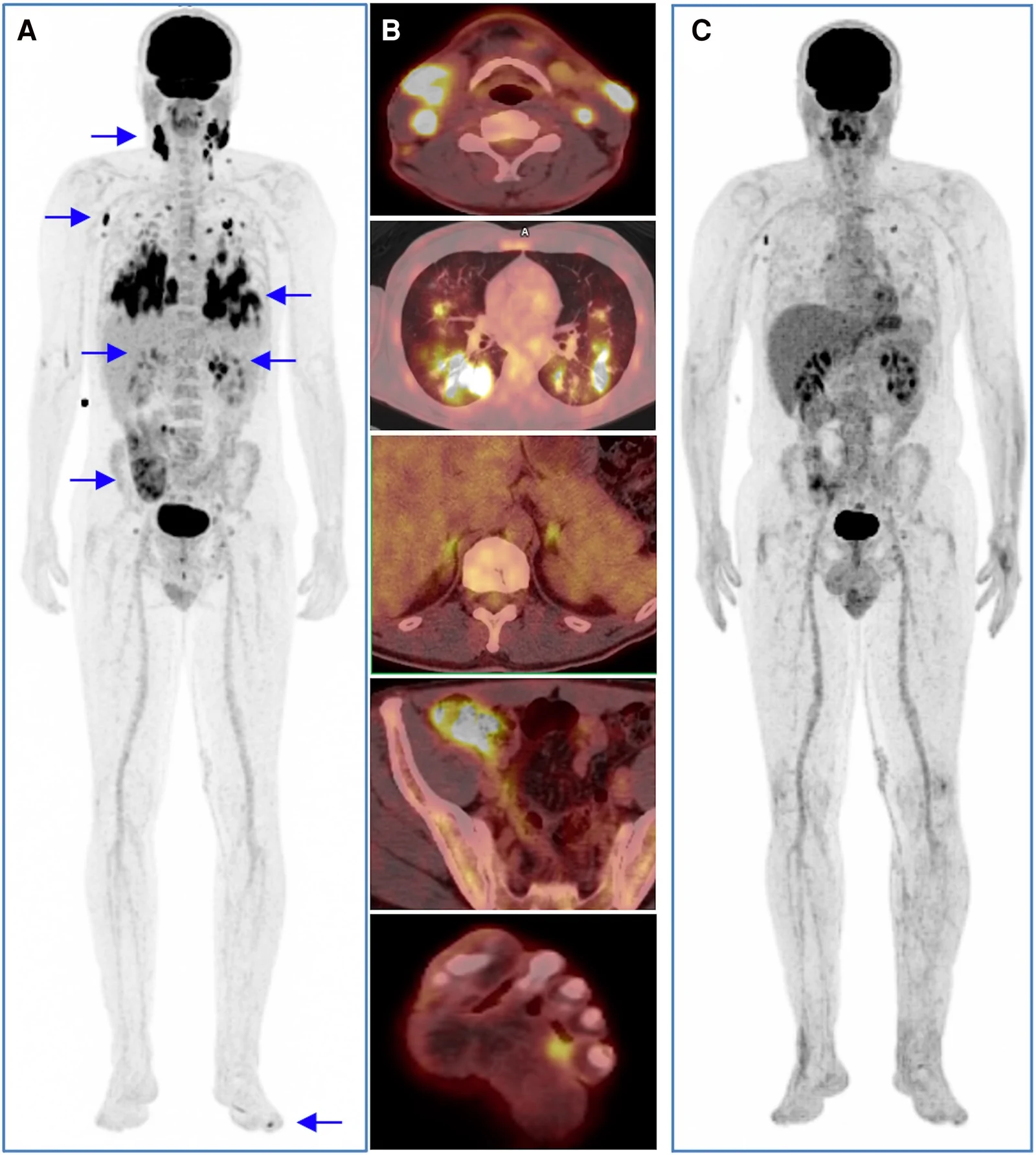
*A*, Whole-body maximum-intensity-projection (MIP) PET image showing multiple FDG-avid foci in a 52-y-old male with disseminated paracoccidioidomycosis (arrows). Clinically, only skin and lymph node involvement had been detected. *B*, Axial fused PET/CT images (from top to bottom) demonstrated extensive involvement of cervical lymph nodes, lungs, adrenal glands, cecum, and a skin lesion in the left foot (arrows). *C*, Six months after treatment with itraconazole, follow-up PET/CT shows marked reduction in FDG uptake in all lesions.

**Figure 2.**
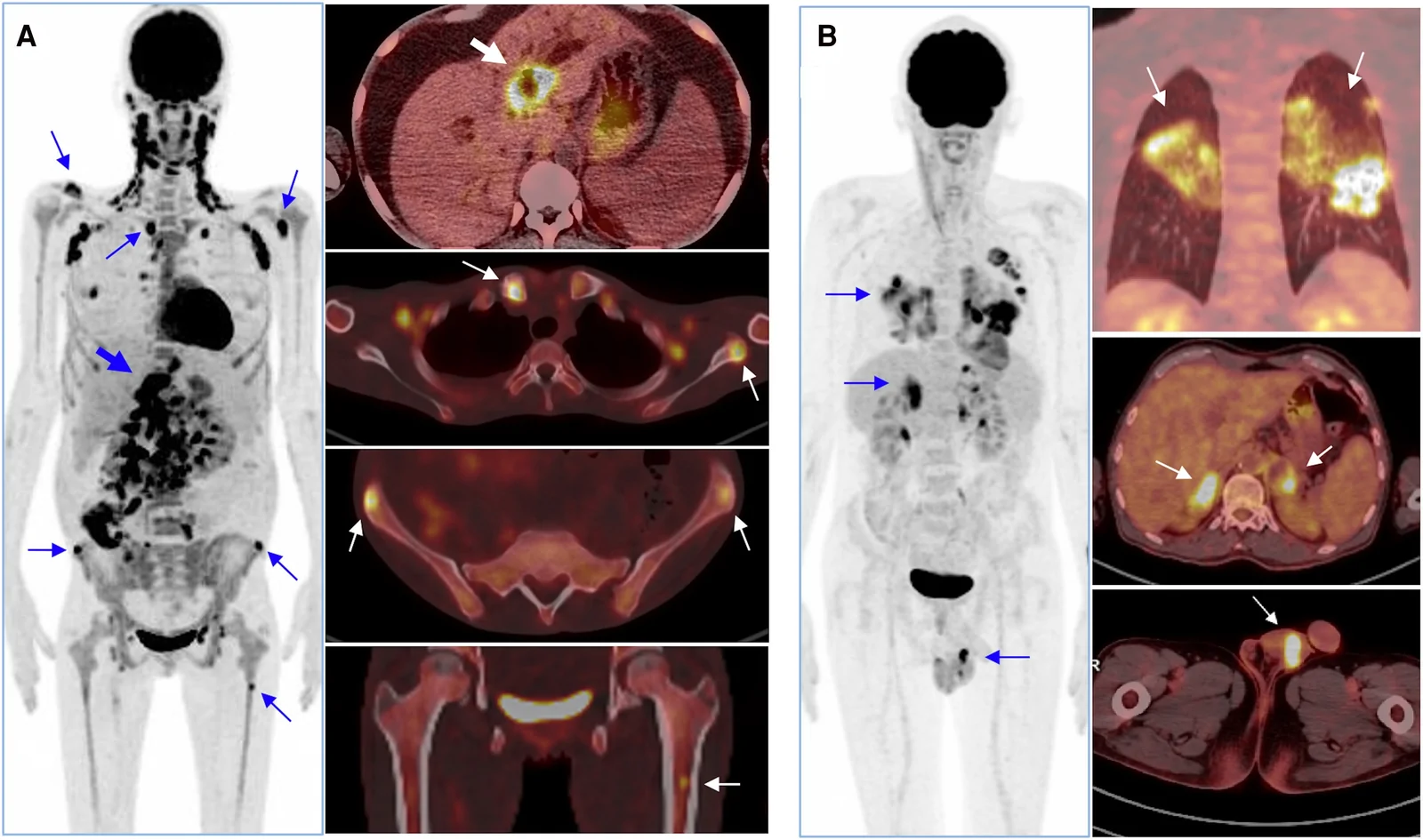
*A*, Hepatic and multiple bone lesions in a 35-y-old female patient admitted with obstructive jaundice and ultimately diagnosed with the juvenile form of paracoccidioidomycosis. MIP (left) and axial fused PET/CT images (right) show multiple small focal lesions in the right clavicle, left scapula, both iliac bones, and the proximal left femur (thin arrows), none of which had been clinically detected. An infiltrative hepatic lesion is also observed (thick arrows). The MIP image additionally reveals involvement of cervical, mediastinal, axillary, and abdominal lymph nodes and physiological cardiac uptake. *B*, Pulmonary, adrenal, and testicular lesion (arrows) are seen in a 58-y-old man presenting with prolonged fever, cough, adrenal insufficiency, and *Paracoccidioides* sp. identified in sputum. Lesions are shown on MIP images (left) and fused PET/CT images (right).

**Figure 3.**
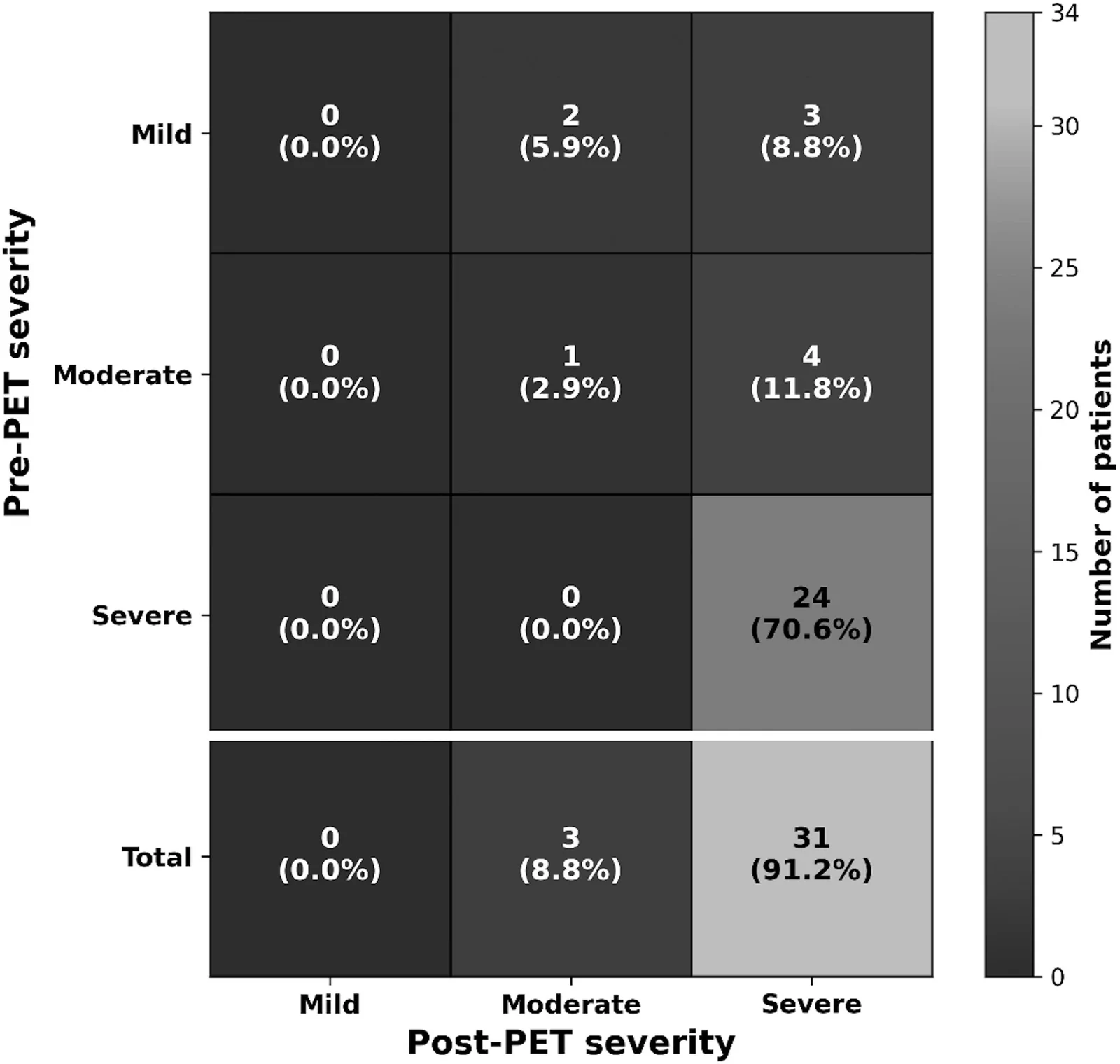
Heatmap representing the reclassification of disease severity after FDG-PET/CT evaluation in 34 patients with paracoccidioidomycosis. Rows indicate pre-PET clinical severity classification (mild, moderate, or severe); columns indicate post-PET severity classification. Cell values indicate the number and percentage of patients in each category. Reclassification was unidirectional, with seven of ten clinically non-severe patients upgraded to severe and no patient downgraded (McNemar's exact test, *P* = .016).

**Table 2.** Comparison Between Conventional Clinical Evaluation and PET/CT

	Total number of affected sites (number of lesions)		Presence of affected sites (number of patients)		
	*Conventional*	*PET/CT*	*Conventional*	*PET/CT*	*P-value*
**Lymph node chains**	17^a^	67^a^	13 (38%)	25 (74%)	.**001**
**Lungs**	9	18	9 (26%)	18 (53%)	.**008**
**Adrenal glands**	5	19^b^	3 (9%)	14 (41%)	.**003**
**Oral mucosa/larynx**	17	17	17 (50%)	17 (50%)	**N/A**
**Bones**	3	6	3 (9%)	6 (18%)	.248
**Skin/subcutaneous**	7	9	7 (21%)	9 (26%)	.480
**Bowel**	1	4	1 (3%)	4 (12%)	.248
**Thyroid**	0	4	0 (0%)	4 (12%)	.134
**Testicles**	0	3	0 (0%)	3 (13%)^c^	.248
**Liver**	2	2	2 (6%)	2 (6%)	**N/A**
**Spleen**	1	1	1 (3%)	1 (3%)	**N/A**
TOTAL	62	150	34 (100%)		

N/A, McNemar test not applicable, no discordant pairs. Bold values indicate statistically significant differences (*P* < .05).

^a^Lymph node chain was divided in cervical, axillary, thoracic, abdominal, and pelvic/inguinal chains.

^b^Bilateral involvement was considered as two affected sites.

^c^Considering only male patients.

Lymph nodes were the most frequently involved additional sites. FDG-PET/CT detected 67 lymph node chains versus 17 by conventional imaging, particularly in thoracic, axillary, abdominal, and pelvic/inguinal regions. Cervical lymph nodes were the most frequently identified by conventional method.

Pulmonary involvement was detected in 18 patients by PET/CT versus 9 by conventional evaluation, with PET/CT revealing more extensive disease in overlapping cases.

Adrenal lesions were observed in 14 patients by PET/CT, compared with only 3 detected by conventional evaluation. In three patients, PET detected lesions without corresponding anatomical changes on CT, highlighting the sensitivity of metabolic imaging. The most common anatomical finding in the CT part of the PET/CT images of these patients was unspecific thickening of adrenal glands which could have been missed without the correlation with PET findings (Figures 1–3).

Focal bowel uptake was observed in four patients, correlating with clinical symptoms in two cases; one asymptomatic lesion was confirmed by colonoscopy. Thyroid uptake was observed in four patients (focal in three, diffuse in one), likely reflecting PCM involvement, although biopsies were not performed.

Inferential analysis using McNemar's test demonstrated statistically significant differences between FDG-PET/CT and conventional staging in the detection of lymph node chain involvement (*P* = .001), pulmonary involvement (*P* = .008), and adrenal gland involvement (*P* = .003). PET/CT also detected more cases of bone, bowel, thyroid, testicular, skin, and subcutaneous involvement than conventional evaluation; however, these differences did not reach statistical significance (*P* = .134–.480), likely owing to the limited number of patients affected at these sites. Complete concordance between methods was observed for the liver, spleen, oral mucosa, and larynx, for which McNemar's test was not applicable (Table 2).

Severity-based reclassification after FDG-PET/CT was unidirectional: no patient was downgraded, while seven of ten clinically non-severe patients (70%) were reclassified as severe, resulting in an overall increase in the proportion of severe cases from 70.6% to 91.2% (McNemar's exact test, *P* = .016). Agreement between clinical and PET-based severity classification was low-to-moderate (quadratic weighted kappa κ = 0.29; 95% CI: 0.00–0.59), consistent with systematic underestimation of disease extent by conventional clinical assessment (Table 3, Figure 3).

**Table 3.** Distribution of Patients with Paracoccidioidomycosis (PCM) According to Disease Severity Classification Before (Clinical Criteria) and After Incorporation of PET/CT Findings

Severity	Before PET/CT	After PET/CT
Mild	5	0
Moderate	5	3
Severe	24	31
**Total**	**34**	**34**

### FDG-PET/CT vs Gallium-67 Scintigraphy

Eight patients underwent gallium-67 scintigraphy for staging. PET/CT was positive in all cases, identifying 35 sites of involvement, whereas gallium-67 detected only 10 sites in 6 patients and was negative in 2. PET/CT revealed adrenal, lymph node, thyroid, larynx, and pulmonary lesions that were missed by gallium-67 (Figure 4).

**Figure 4.**
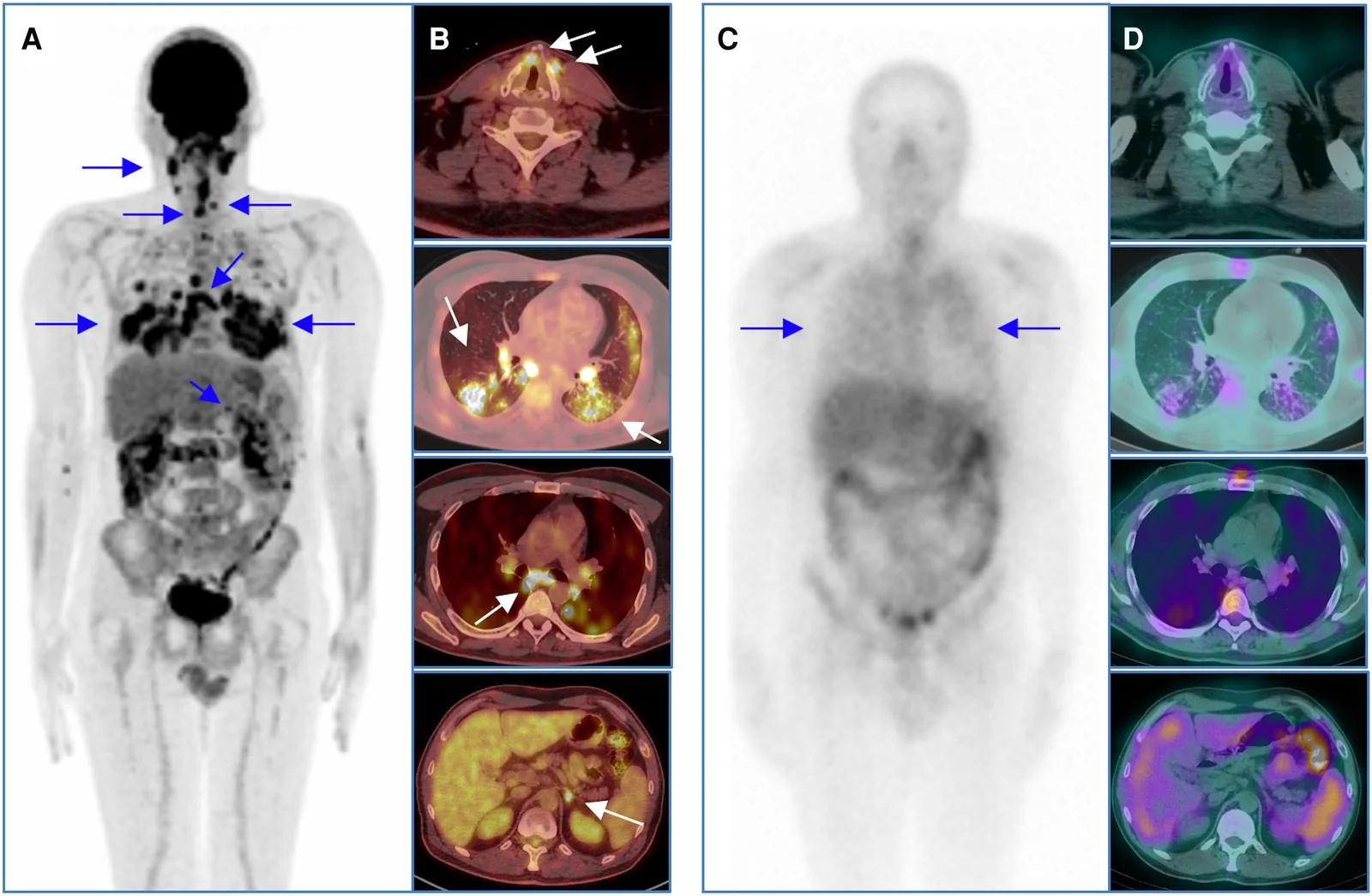
*A*, MIP PET image showing multiple FDG-avid foci in a 40-y-old male with paracoccidioidomycosis confirmed by pathological examination (arrows). *B*, Axial fused PET/CT images (from top to bottom) demonstrate focal FDG uptake in the larynx, extensive pulmonary involvement—predominantly in the lower lobes—a subcarinal lymph node, and the left adrenal gland (arrows). *C*, Anterior planar gallium-67 scintigraphy performed 7 days later shows only mild diffuse pulmonary uptake (arrows). *D*, Axial fused gallium-67 SPECT/CT images displayed at the same levels as in (*B*) show no tracer uptake in the larynx, the subcarinal lymph node, or the left adrenal gland and show only mild uptake in focal pulmonary consolidations.

### Quantitative PET/CT Analysis

The mean whole-body SUVmax was 12.4 ± 6.8 (range 2.5–26.9). Mean TMLV and TLG were 164.0 ± 233.6 and 713.5 ± 1089.5, respectively. Quantitative values for organ-specific involvement are summarized in Table 4.

**Table 4.** Quantitative FDG-PET/CT Data in PCM Patients

Variable	Mean ± SD; median (min–max)
**SUVmax (whole-body)**	12.4 ± 6.8; 12.2 (2.5–26.9)
**TMLV [ml]**	164.0 ± 233.6; 69.9 (0.0–1062.2)
**TLG**	713.5 ± 1089.5; 252.5 (0.0–4670.1)
**SUVmax lung**	8.8 ± 6.4; 6.6 (2.7–26.9)
**SUVmax lymph nodes**	11.7 ± 6.2; 10.6 (2.5–23.0)
**SUVmax adrenal**	7.9 ± 4.4; 7.9 (2.6–16.7)
**SUVmax bone**	12.5 ± 6.9; 9.6 (6.3–23.2)
**SUVmax subcutaneous nodule**	5.7 ± 1.9; 6.3 (2.6–8.0)

SUVmax, maximum standardized uptake value; TLG, total lesion glycolysis; TMLV, total metabolic lesion volume.

DTPI performed in 15 patients demonstrated higher delayed uptake, with mean SUVmax increasing from 9.5 ± 6.3 to 12.8 ± 7.4, corresponding to a mean SUVvar of 44.9 ± 34.5%.

### Correlation with Serology and Inflammatory Markers

Patients were grouped by serological titers: negative (0), mildly abnormal (1:2–1:8), and moderate/severely abnormal (1:16–1:256). Erythrocyte sedimentation rate values ranged from 2 to 120 mm (mean 49.9 ± 39.1), and CRP levels ranged from 0.10 to 89.7 mg/L (mean 13.5 ± 19.0).

Patients with negative serology exhibited lower SUVmax, TMLV, and TLG compared to those with mildly or moderately/severely abnormal serology (*P* < .05, Kruskal–Wallis test with Dunn's post hoc test) (Table 5, Figure 5). Notably, 8 of 34 patients (24%) had nonreactive serology despite active lesions identified on FDG-PET/CT.

**Figure 5.**
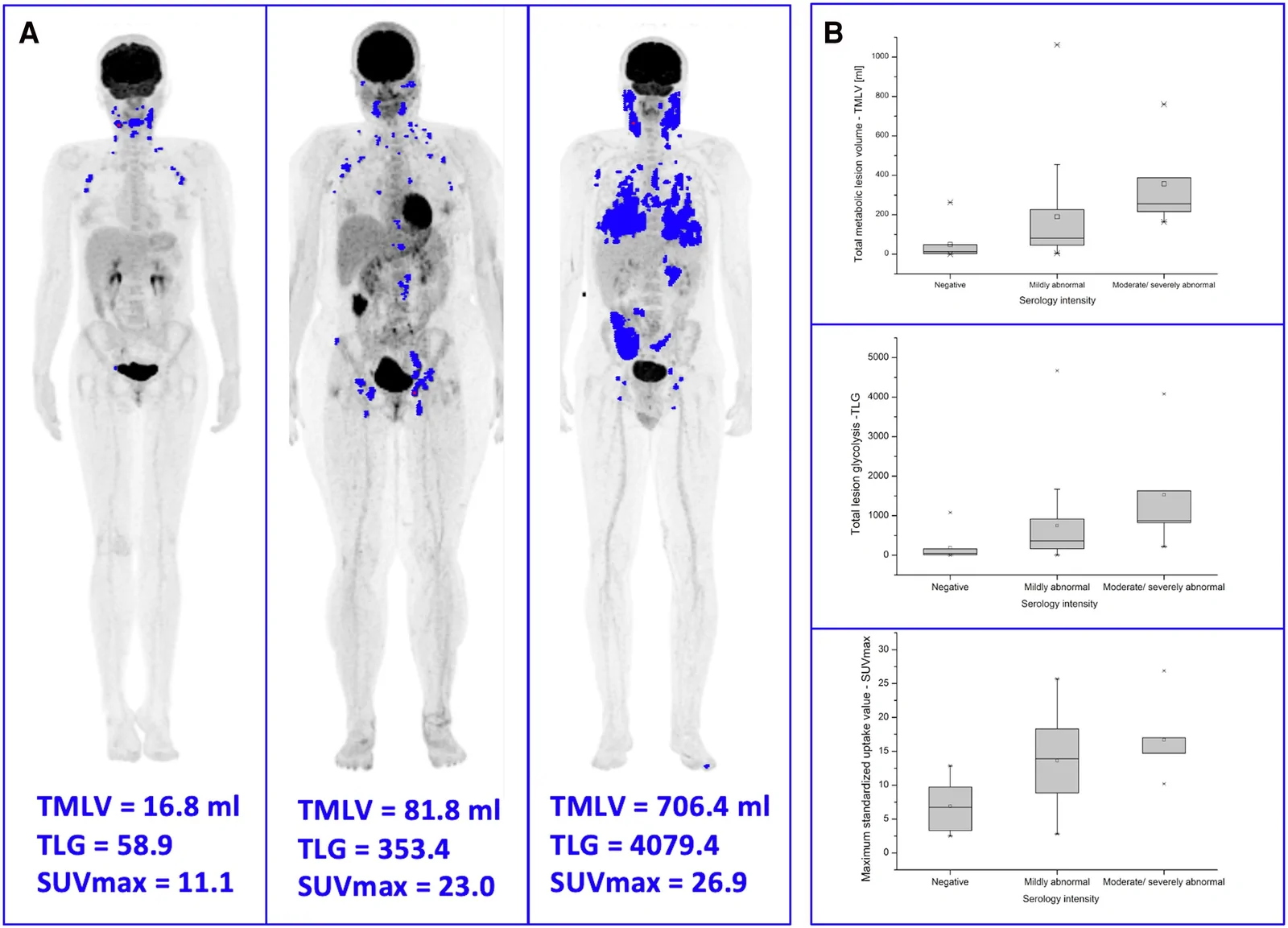
*A*, From left to right, patients with serology classified as negative, mildly abnormal, and moderately/severely abnormal are shown, along with their corresponding total metabolic lesion volume (TMLV), total lesion glycolysis (TLG), and maximum standardized uptake value (SUVmax). Paracoccidioidomycosis lesions were automatically segmented in blue using free software for disease-burden quantification. *B*, Boxplots illustrating the distribution of TMLV (top), TLG (middle), and SUVmax (bottom) according to serological classification (negative, mildly abnormal, and moderately/severely abnormal). A marked difference is observed between the negative group and the other groups.

**Table 5.** SUVmax, TMLV, and TLG by Serological Test Reactivity Groups

	Negative serology (n = 8) Median [range]	Mildly abnormal serology^a^ (n = 20) Median [range]	Moderate/severely abnormal serology^b^ (n = 5) Median [range]	*P-v*alue^c^
SUVmax	6.7 [2.5–12.9]	13.9 [2.8–25.7]	14.7 [10.2–26.9]	.0153
TMLV	12.6 [0–262.6]	89.0 [0.2–1062.2]	212.5 [64.0–706.5]	.0146
TLG	42.0 (0–1080.7)	361.7 [0.7–4670.1]	876.3 [215.3–4079.4]	.0146

SUVmax, maximum standardized uptake values; TLG, total lesion glycolysis; TMLV, total metabolic lesion volume.

^a^Antibody titers from 1:2 to 1:8.

^b^Antibody titers from 1:16 to 1:256.

^c^Kruskal–Wallis test.

Significant correlations were observed between inflammatory markers and whole-body PET/CT quantifications. C-reactive protein demonstrated a moderate positive correlation with whole-body SUVmax (*r* = 0.452, *P* = .0073), TMLV (*r* = 0.450, *P* = .0076), and TLG (*r* = 0.477, *P* = .0043) and a strong correlation with pulmonary SUVmax (*r* = 0.686, *P* = .0024). Erythrocyte sedimentation rate also correlated positively with SUVmax (*r* = 0.343, *P* = .0470), TMLV (*r* = 0.407, *P* = .0170), and TLG (*r* = 0.392, *P* = .0218), with the correlation between ESR and adrenal SUVmax being particularly high (*r* = 0.589, *P* = .0265).

Patients with suspected treatment failure showed significantly lower TMLV and TLG values compared with treatment-naïve patients (median TMLV: 40.9 vs 86.0 mL; median TLG: 169.5 vs 489.7; Mann–Whitney *U* test, *P* < .05), whereas no significant difference in SUVmax was observed between the two groups.

### PET/CT and Demographics

Adrenal involvement was exclusively observed in males (*P* = .0007) and was age-related, with affected patients being older (median 58.5 years vs 42 years, *P* = .0008). Patients with lymph node involvement were younger than those without (mean 48 vs 63 years, *P* = .0172), with age negatively correlating with TMLV (*r* = −0.4182, *P* = .0139), TLG (*r* = −0.4359, *P* = .0100), and SUVmax (*r* = −0.4782, *P* = .0156) in lymph nodes.

## DISCUSSION

This study highlights the superior sensitivity of FDG-PET/CT, detecting more than twice the number of active PCM lesions compared with conventional clinical evaluation. The significant and unidirectional post-PET shift toward severe disease classification supports the concept that PCM is frequently more disseminated than suggested by clinical assessment alone. FDG-PET/CT should therefore be interpreted not as a substitute for established clinical severity criteria but as a complementary staging tool capable of identifying patients whose true disease burden exceeds what conventional evaluation reveals—with direct implications for treatment duration and follow-up intensity.

The study also suggests that real cases of unifocal disease are likely to be exceptions. Although unifocal PCM has been described in at least 25% of patients, generally as pulmonary disease [25], using PET/CT only one patient remained unifocal and 97% of the patients were identified with multifocal disease. The sensitivity to determine disease extension was also supported by the finding that inflammatory markers correlated with PET/CT quantifications, including SUVmax, TMLV, and TLG. The observed difference in TMLV and TLG between treatment-naïve patients and those with suspected treatment failure—with no corresponding difference in SUVmax—suggests that antifungal therapy reduces the overall extent of metabolically active disease while individual lesions may retain significant FDG avidity, potentially reflecting persistent infection and treatment-refractory foci. This dissociation between total disease burden and lesion-level metabolic activity has potential implications for the interpretation of PET/CT findings in patients undergoing treatment and warrants further prospective investigation.

Pulmonary involvement in our series was lower than that described in earlier clinical or necropsy studies [5, 14]. A plausible explanation is that pulmonary PCM may respond more effectively to initial treatment than involvement of other organs. Nevertheless, FDG-PET/CT revealed pulmonary disease in twice as many patients compared with conventional evaluation, suggesting that the functional technique uncovers a broader spectrum of pulmonary activity.

FDG-PET/CT also demonstrated lymph node involvement in 74% of patients, a prevalence remarkably similar to the 72% reported by Salfelder et al in necropsy studies [15] but much higher than the ∼50% described in large clinical cohorts such as that of Belíssimo-Rodrigues et al [14]. This discrepancy indicates that nodal disease is often underdiagnosed in routine practice. As in oncology, PET/CT appears capable of detecting nodal lesions below the threshold of conventional imaging, approaching the sensitivity of postmortem studies.

One of the most striking findings was the high prevalence of adrenal involvement (44%), a result previously documented only in autopsy series, where frequencies ranged from 36% to 80% [4, 15]. By contrast, large clinical studies reported adrenal disease in only 2% to 5% of cases [14, 26]. Since adrenal insufficiency is a major cause of mortality in PCM [26], early detection of adrenal involvement by PET/CT has direct clinical relevance. Moreover, our observation of a predominance in older men has not been previously reported and warrants further investigation.

Bone disease was identified in 18% of patients, a figure closer to necropsy reports [27] than to clinical series, which often fail to document skeletal involvement [14]. Similarly, testicular lesions were detected in 9% of patients, again paralleling autopsy findings [27]. These results highlight the ability of PET/CT to reproduce, in vivo, the spectrum of organ involvement traditionally seen only at necropsy.

Thyroid uptake was observed in 12% of patients, without CT-detectable anatomical lesions. Although nonspecific, FDG uptake may represent PCM involvement, which has been incidentally described at autopsy [15]. Endocrine dysfunction related to PCM has been reported, particularly altered T4-to-T3 conversion in severe or juvenile forms [11, 28], and deserves further investigation.

Intestinal involvement (12%) was also more frequent than previously recognized clinically, where diagnosis is often hindered by nonspecific symptoms [29]. Paracoccidioidomycosis should therefore be considered in the differential diagnosis of inflammatory bowel diseases in endemic regions [30]. Still, in non-biopsy-confirmed cases, the possibility of false-positive uptake from inflammatory conditions must be acknowledged.

Importantly, a quarter of patients had negative serology but positive FDG-PET/CT, confirming that PET is more sensitive for detecting active disease. Quantitative PET metrics correlated both with serology and with inflammatory markers, reinforcing their role as objective markers of disease burden. These metrics, widely used in oncology for therapy monitoring, could potentially be adapted to evaluate antifungal treatment response in PCM, as previously suggested for other systemic mycoses [21]. In a small subset of patients with documented clinical response to treatment, follow-up FDG-PET/CT demonstrated marked reduction in tracer uptake across previously involved sites (Figure 1*C*), supporting the potential utility of this modality for monitoring treatment response in PCM.

Dual-time-point imaging yielded increasing SUVmax values in all PCM lesions, mimicking the kinetics of malignant disease. While intriguing, this finding indicates that DTPI does not help distinguish PCM from cancer, limiting its clinical utility.

Compared to gallium-67 scintigraphy, previously regarded as the functional gold standard in PCM [31, 32], FDG-PET/CT was clearly superior. It not only detected more lesions but also identified cases missed by gallium imaging while providing superior image quality and same-day results.

By revealing a higher burden of active disease than conventional staging, FDG-PET/CT may support more individualized therapeutic decisions in PCM, including prolonged antifungal treatment in patients with extensive or occult organ involvement, and closer follow-up of critical sites—particularly the adrenal glands—where persistent infection may predispose to irreversible insufficiency or late reactivation.

The present study has limitations. Conventional staging in this cohort was not uniform, as additional imaging beyond chest radiography was performed selectively based on clinical suspicion—reflecting standard practice in PCM rather than a predefined protocol. This heterogeneity should be considered when interpreting comparisons with FDG-PET/CT, which was applied systematically to all patients. Additionally, histopathological confirmation was not obtained for every PET-detected lesion, as biopsy of all involved sites was neither clinically indicated nor ethically justified; consequently, the possibility of false-positive findings due to overlap with other infectious, inflammatory, or neoplastic conditions cannot be entirely excluded. The cohort of 34 patients, while appropriate given the rarity and clinical heterogeneity of PCM and the logistical demands of prospective whole-body PET/CT acquisition, limits broader generalizability. Finally, the absence of systematic follow-up imaging constrains conclusions regarding the utility of FDG-PET/CT for treatment monitoring, an application that warrants dedicated prospective investigation.

Although PET/CT infrastructure has expanded considerably across Latin America—a region where the disease is endemic—its use remains almost exclusively restricted to oncological indications, and access is concentrated in urban private centers far from the rural populations most affected by PCM [33, 34]. Additional barriers include cost and radiation exposure. Until broader access and dedicated reimbursement policies are established, PET/CT for PCM staging may be best reserved for cases where accurate disease mapping would directly change clinical management—underscoring that neglected diseases require not only new drugs but also modern imaging technologies.

## CONCLUSIONS

Taken together, these findings support a paradigm shift in the staging of PCM: from a symptom–and organ–driven assessment limited by the sensitivity of conventional tools toward a whole-body metabolic evaluation capable of revealing the true extent of systemic involvement. By systematically reclassifying patients toward higher severity categories and identifying occult organ involvement with direct prognostic implications, FDG-PET/CT has the potential to refine the initial staging of PCM and to support more individualized therapeutic decisions—particularly regarding treatment duration and intensity of organ-specific follow-up. Its role in treatment monitoring, while biologically plausible and supported by preliminary observations, remains to be formally established in prospective studies.
